# MicroRNA-451 inhibits inflammation and proliferation of glomerular mesangial cells through down-regulating PSMD11 and NF-κB p65

**DOI:** 10.1042/BSR20191455

**Published:** 2019-10-25

**Authors:** Hua Wei, Jianzhou Li, Yanhua Li, Jian Song

**Affiliations:** 1 Department of Endocrinology, Shouguang People’s Hospital, No. 1233, Jiankang Street, Shouguang City, Shandong Province 262700, China; 2 Department of Endocrinology, Caoxian People’s Hospital, East Qinghe Road, South Fumin Avenue, Caoxian Development Zone, Heze City 274400, Shandong Province, China; 3 Department of Medical, The First People’s Hospital of Jinan City, No. 132, Daminghu Road, Lixia District, Jinan City 250011, Shandong Province, China; 4 Department of Nephrology, Qilu Hospital of Shandong University, No. 107, Wenhua West Road, Jinan City 250012, Shandong Province, China

**Keywords:** High-glucose, Inflammation, MicroRNA-451, Nuclear factor-kappa B, Proliferation

## Abstract

The present study aimed to investigate the regulatory roles of microRNA-451 (miR-451) on the inflammation and proliferation of glomerular mesangial cells (GMCs) under high-glucose condition, and reveal the potential mechanisms related to 26S proteasome non-ATPase regulatory subunit 11 (PSMD11) and nuclear factor-κ B (NF-κB) signaling. The interaction between PSMD11 and miR-451 was identified by dual luciferase reporter (DLR) gene assay. GMCs were treated with 5.6 mmol/l (normal, L-GMCs) and 30 mmol/l glucose (high-glucose, H-GMCs), respectively. After transfecting with pcDNA3.1-PSMD11 and/or miR-451 mimics, the expression of miR-451, PSMD11, inhibitor of NF-κB α (IκBα), phosphorylated IκBα (p-IκBα), NF-κB p65, COX-2, and cyclinD1 were detected in H-GMCs by quantitative real-time PCR (qRT-PCR) and/or Western blot. The levels of interleukin (IL)-1β, IL-6, and IL-8, cell cycle, and viability was detected by enzyme-linked immunosorbent assay, flow cytometry, and MTT assay, respectively. MiR-451 was up-regulated in H-GMCs, and negatively regulated its target PSMD11 (*P*<0.05). H-GMCs exhibited significantly higher levels of IL-1β, IL-6, and IL-8, cell viability, and p-IκBα, NF-κB, COX-2, and cyclinD1 expression than L-GMCs (*P*<0.05). The transfection of miR-451 mimics significantly decreased the levels of IL-1β, IL-6, and IL-8, inhibited the cell viability via blocking cells in G_0_/G_1_ phase, and down-regulated p-IκBα, NF-κB p65, COX-2, and cyclinD1 in H-GMCs (*P*<0.05). The regulatory effects of miR-451 mimics on H-GMCs were reversed by the transfection of PSMD11 (*P*<0.05). The up-regulation of miR-451 inhibits the inflammation and proliferation of H-GMCs through down-regulating PSMD11 and NF-κB p65.

## Introduction

Diabetic nephropathy (DN) is a common disease that occurs in approximately 30% of type 2 diabetic patients [1]. DN is mainly characterized by mesangial proliferation, glomerular basement membrane thickening, and podocyte loss [2,3]. In clinical practice, glycemic control, blood pressure control, and renoprotection are the main therapeutic strategies for DN [4]. With increasing knowledge of the pathogenesis of DN, molecular targeting therapy has become a promising therapeutic strategy for DN [5].

MicroRNAs (miRs) are important post-transcriptional regulators in DN [6]. A previous study has proved that miR-192, -216a, -217, -21, -377, -195, -215, -124, -29c, and -135a are up-regulated, and miR-200a, -29a/b, -451, -25, and -93 are down-regulated in DN [7]. MiR-192, -29c, -135a, and -21 are considered as the therapeutic targets for DN [8]. MiR-451 also plays a key regulatory role in DN [9–11]. It has been reported that microRNA-451 (miR-451) expression is decreased in the kidney, peripheral blood mononuclear cells (PBMCs), as well as glomerular mesangial cells (GMCs) under high-glucose condition [9,10]. The up-regulation of miR-451-5p protects against diabetes-induced kidney fibrosis in DN rats [11]. However, researches on the specific regulatory roles and mechanisms of miR-451 on GMCs are still limited.

Nuclear factor-κ B (NF-κB) is activated in DN patients, experimental animal models of DN, and GMCs under high-glucose condition [12,13]. Various potential therapeutic agents have been identified to ameliorate DN via inhibiting NF-κB, such as caprylic acid-diacylglycerol oil [14], ellagic acid [15], and curcumin [16]. In addition, a previous study has proved that miR-451 inhibits NF-κB activity via targeting large multifunctional protease 7 (LMP7), thereby down-regulating pro-inflammatory molecules in mesangial cells [9]. However, the regulatory mechanisms of miR-451 related to NF-κB are still not fully revealed on GMCs.

The 26S proteasome non-ATPase regulatory subunit 11 (PSMD11) is a multicatalytic proteinase complex that plays a key regulatory role in proteasome activity in embryonic stem cells [17]. Until now, the knowledge on the regulatory roles of PSMD11 on DN is limited. In the present study, a specific interaction between PSMD11 and miR-451 was first identified. Then the regulatory roles of miR-451 on the inflammation and proliferation of GMCs under high-glucose condition were evaluated. Furthermore, the regulatory mechanisms of miR-451 relating with PSMD11 and NF-κB were analyzed. Our findings may reveal a novel therapeutic target for DN, and open up new insights into the underlying mechanisms of DN.

## Methods

### Cell culture and treatments

GMCs (SV40-MES-13) were purchased from The Cell Bank of Chinese Academy of Sciences (Shanghai, China). Cells were cultured in Dulbecco’s Modified Eagle’s Medium (DMEM) containing 10% fetal bovine serum (FBS), and maintained in a humidified incubator at 37°C with 5% CO_2_. Cells were passaged until 80–90% confluence. GMCs at the third passage were randomly divided into two groups, including L-GMCs (normal physiological environment, 5.6 mmol/l glucose) and H-GMCs (high-glucose environment, 30 mmol/l glucose).

### Cell transfection

MiR-451 mimics, miR-451 mimics negative control (mimics NC), and pcDNA3.1-PSMD11 were purchased from Guangzhou Ruibo Biotechnology Co., Ltd. (Guangzhou, China). GMCs in logarithmic growth phase were seeded in six-well plates, and then transfected with miR-451 mimics, mimics NC, pcDNA3.1-PSMD11, and miR-451 mimics + pcDNA3.1-PSMD11 using Lipofectamine 2000 (Thermo Fisher Scientific, Waltham, MA, U.S.A.), respectively. The transfected cells were used for further assays after the transfection for 48 h.

### Quantitative real-time PCR

Total RNA was extracted from GMCs using TRIzol, and cDNA was reverse-transcribed using a cDNA Reverse Transcription Kit (Thermo Fisher Scientific) in accordance with manufacturers’ instructions. Quantitative real-time PCR (qRT-PCR) was performed by using specific primers (miR-451-F, 3′-CCGAAACCGTTACCATTAC-5′; miR-451-R, 3′-GTGCAGGGTCCGAGGT-5′; PSMD11-F, 3′-AGTTCCAGAGAGCCCAGTCT-5′; PSMD11-R, 3′-TTGCACTGCCTCTTCATCGT-5′) on ABI 7500 Real-Time PCR System (Applied Biosystems, Foster City, CA, U.S.A.). GAPDH was used as an internal control (GAPDH-F, 3′-TGACTTCAACAGAGACACCA-5′; GAPDH-R, 3′-GCTGTTGGGCTGTAGGGA-5′). The PCR program included 95°C for 10 min, 40 cycles of 95°C for 10 s, 60°C for 20 s, and 72°C for 34 s. The relative expression level was calculated according to the 2^−ΔΔ*C*_t_^ method [18].

### Western blot

GMCs were lysed in RIPA lysis buffer (Beyotime, Shanghai, China). Total proteins were separated by sodium dodecyl sulfate/polyacrylamide gel electrophoresis, transferred to polyvinylidenefluoride membrane, and blocked with 5% skim milk for 2 h. Then the membrane was incubated with specific primary antibodies (rabbit anti-mouse; anti-PSMD11, #14303; anti-proliferating cell nuclear antigen (PCNA), #13110; anti-inhibitor of NF-κB α (IκBα), #4812; anti- phosphorylated IκBα (p-IκBα), #2859; anti-NF-κB p65, #8242; anti-Histone H3, #9728; anti-COX-2, #4842; anti-cyclinD1, #2922; anti-GAPDH, #5174; 1:1000, Cell Signaling Technology, Boston, U.S.A.) for 12 h at 4°C. After incubating with horseradish peroxidase (HRP)–conjugated secondary antibody (goat anti-rabbit; 1:2000, Cell Signaling Technology) for 1 h at 25°C, the protein brands were visualized using an HRP color development kit (Thermo Fisher Scientific). The protein level was normalized to GAPDH, and standardized to L-GMC group (set as 1).

### Dual luciferase reporter gene assay

HEK-293T cells (Cell Bank of Chinese Academy of Sciences) were co-transfected with pGL3 luciferase plasmids (Promega, Madison, WI, U.S.A.) carrying PSMD11-wildtype (PSMD11-WT)/PSMD11-mutant (PSMD11-MUT) and miR-451 mimics/mimics NC for 24 h. The fluorescence was visualized using a Dual Luciferase Reporter (DLR) Assay Kit (Promega, Madison, WI, U.S.A.). The fluorescence intensity was detected by using a Microplate Reader (Molecular Devices, Sunnyvale, CA, U.S.A.).

### Enzyme-linked immunosorbent assay

Interleukin (IL)-1β, IL-6, and IL-8 were detected in GMCs by using Enzyme-linked immunosorbent assay (ELISA) kits (Boster, Wuhan, China) in accordance with manufacturers’ instructions. The optical density (OD) at 450/550 nm was detected by a Microplate Reader (Molecular Devices).

### Flow cytometry

GMCs were fixed in 70% ethanol for 12 h at 4°C, and washed with phosphate buffer saline (PBS). After incubating with Muse Cell Cycle Reagent (Millipore, U.S.A.) in the dark for 30 min, cells were analyzed on MUSE cell analyzer (Millipore).

### MTT assay

GMCs were seeded in 12-well plates, and incubated with MTT (Sigma, U.S.A.) for 4 h. Then the medium was removed, and DMSO was added. The OD at 495 nm was detected by Microplate Reader (Molecular Devices).

### Immunofluorescence

GMCs were fixed in 4% paraformaldehyde for 20 min at 4°C, and blocked with 5% BSA for 30 min. Then cells were incubated with primary antibody (anti-Ki67, #9449, 1:500, Cell Signaling Technology) overnight at 4°C. After three times of washing with PBS, cells were incubated with Alexa Fluor 594–conjugated secondary antibody (1:500, Cell Signaling Technology) for 1 h at 37°C, and then stained with 4,6-diamino-2-phenylindole (DAPI). Stained cells were observed under fluorescence microscope (Olympus).

### Statistical analyses

All data were expressed as mean ± standard deviation. Comparison between different groups was determined by *t* test (two groups) or one-way ANOVA (>two groups) using SPSS version 21.0 (SPSS Inc., Chicago, IL, U.S.A.). A *P*-value < 0.05 represented significant difference.

## Results

### PSMD11 was a target of miR-451

**Figure 1:**
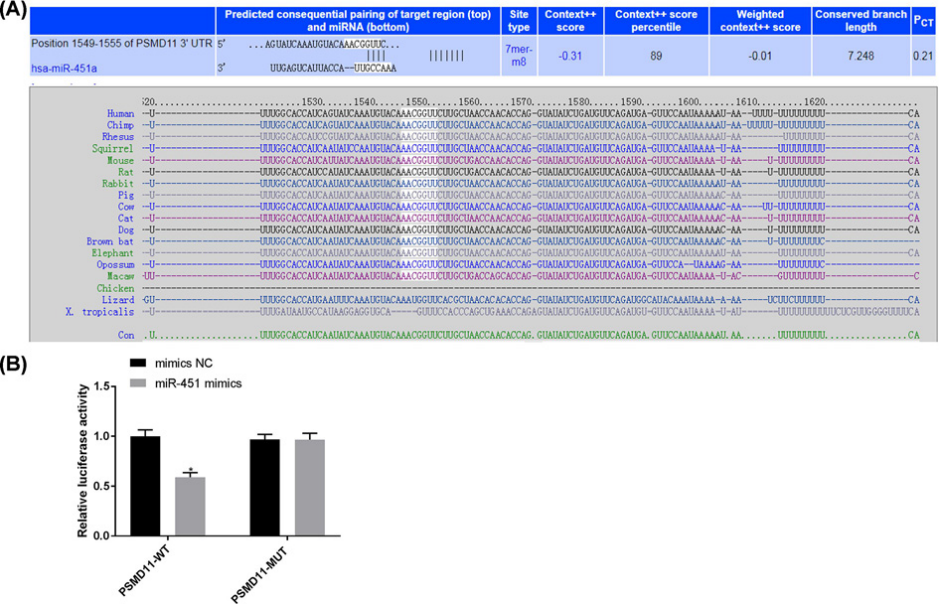
The interaction between miR-451 and PSMD11 (**A**) A binding site of miR-451 at 3′-UTR of PSMD11 predicted by the software of TargetScan. (**B**) Relative fluorescence intensity of HEK-293T cells co-transfected with PSMD11-WT (COL1A1-WT)/ PSMD11-MUT and miR-451 mimics/miR-451 mimics negative control (mimics NC). *P<0.05.

A binding site of miR-451 was predicted at 3′-UTR of PSMD11 by an online target gene prediction software (TargetScan) (Figure 1A). DLR assay showed that the fluorescence intensity was significantly lower in HEK-293T cells co-transfected with PSMD11-WT + miR-451 mimics (0.589 ± 0.047) than those co-transfected with PSMD11-MUT + miR-451 mimics (0.968 ± 0.051), PSMD11-MUT + mimics NC (0.965 ± 0.065), and COL1A1-WT + mimics NC (1.000 ± 0.064) (*P*<0.05) (Figure 1B).

### miR-451 negatively regulated PSMD11 in H-GMCs

**Figure 2:**
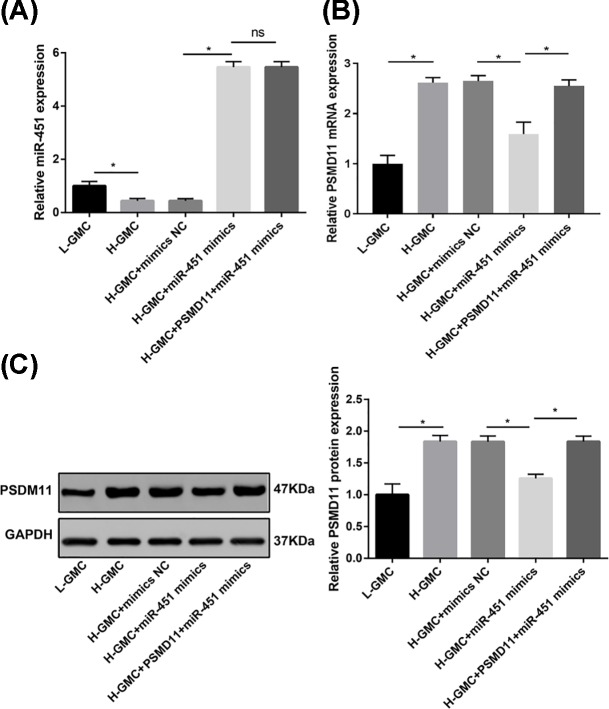
The expression of miR-451 and PSMD11 in GMCs (**A**) The expression of miR-451 detected by qRT-PCR at the mRNA level. (**B**) The expression of PSMD11 detected by qRT-PCR at the mRNA level. (**C**) The expression of PSMD11 detected by Western blot at the protein level. L-GMCs, GMCs treated with 5.6 mmol/l glucose (normal control); H-GMCs, GMCs treated with 30 mmol/l glucose (high glucose); H-GMCs + mimics NC, H-GMCs transfected with miR-451 mimics negative control; H-GMCs + miR-451 mimics, H-GMCs transfected with miR-451 mimics; H-GMCs + PSMD11 + miR-451 mimics, H-GMCs transfected with pcDNA3.1-PSMD11 and miR-451 mimics. *P<0.05.

The expression of miR-451 and PSMD11 were detected in H- and L-GMCs. qRT-PCR showed that miR-451 expression was significantly lower in H-GMCs (0.443 ± 0.085) than in L-GMCs (1.000 ± 0.165) at the mRNA level (*P*<0.05) (Figure 2A). PSMD11 expression was significantly higher in H-GMCs (mRNA, 2.620 ± 0.098; protein, 1.837 ± 0.095) than in L-GMCs (mRNA, 1.000 ± 0.180; protein, 1.000 ± 0.160) at both the mRNA and protein levels (*P*<0.05) (Figure 2B,C). The transfection of miR-451 mimics significantly up-regulated miR-451 (5.463 ± 0.206), and down-regulated PSMD11 (mRNA, 1.593 ± 0.238; protein, 1.257 ± 0.065) (*P*<0.05) in H-GMCs. The down-regulated PSMD11 was recovered by the transfection of PSMD11 in miR-451 mimics-transfected H-GMCs (mRNA, 2.55 ± 0.125; protein, 1.837 ± 0.085) (*P*<0.05). However, miR-451 expression was not significantly influenced by the transfection of PSMD11 in miR-451 mimics-transfected H-GMCs. Both the expression of miR-451 and PSMD11 were not significantly influenced by the transfection of mimics NC (Figure 2A,C).

### Up-regulation of miR-451 inhibited the inflammatory response of H-GMCs

**Figure 3:**
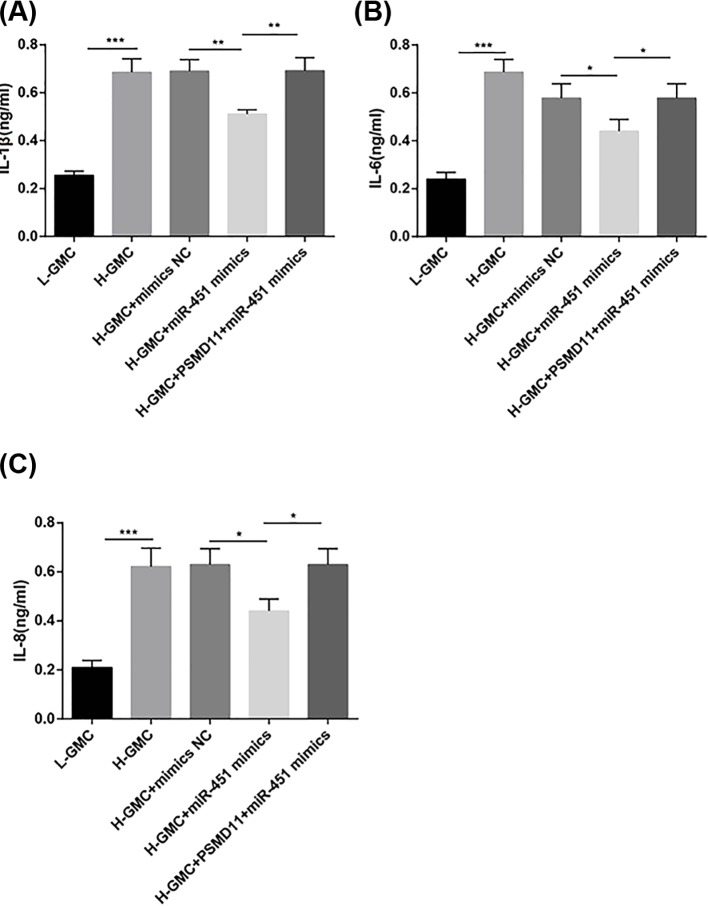
The levels of pro-inflammatory cytokines in GMCs (**A**) IL-1β; (**B**) IL-6; (**C**) IL-8. L-GMCs, GMCs treated with 5.6 mmol/l glucose (normal control); H-GMCs, GMCs treated with 30 mmol/l glucose (high glucose); H-GMCs + mimics NC, H-GMCs transfected with miR-451 mimics negative control; H-GMCs + miR-451 mimics, H-GMCs transfected with miR-451 mimics; H-GMCs + PSMD11 + miR-451 mimics, H-GMCs transfected with pcDNA3.1-PSMD11 and miR-451 mimics. *P<0.05; **P<0.01; ***P<0.001.

The inflammatory response of GMCs was evaluated. As shown in Figure 3A-C, IL-1β, IL-6, and IL-8 levels were significantly higher in H-GMCs (IL-1β, 0.686 ± 0.055; IL-6, 0.687 ± 0.053; IL-8, 0.622 ± 0.075) than in L-GMCs (IL-1β, 0.256 ± 0.017; IL-6, 0.240 ± 0.029; IL-8, 0.210 ± 0.028) (*P*<0.001). The transfection of miR-451 mimics significantly decreased IL-1β, IL-6, and IL-8 levels in H-GMCs (IL-1β, 0.511 ± 0.018; IL-6, 0.440 ± 0.049; IL-8, 0.443 ± 0.056) (*P*<0.01). The increased IL-1β, IL-6, and IL-8 levels were recovered by the transfection of PSMD11 in miR-451 mimics-transfected H-GMCs (IL-1β, 0.691 ± 0.055; IL-6, 0.578 ± 0.059; IL-8, 0.630 ± 0.064) (*P*<0.05). IL-1β, IL-6, and IL-8 levels were not significantly influenced by the transfection of mimics NC (Figure 3A-C).

### Up-regulation of miR-451 inhibited the proliferation of H-GMCs

**Figure 4:**
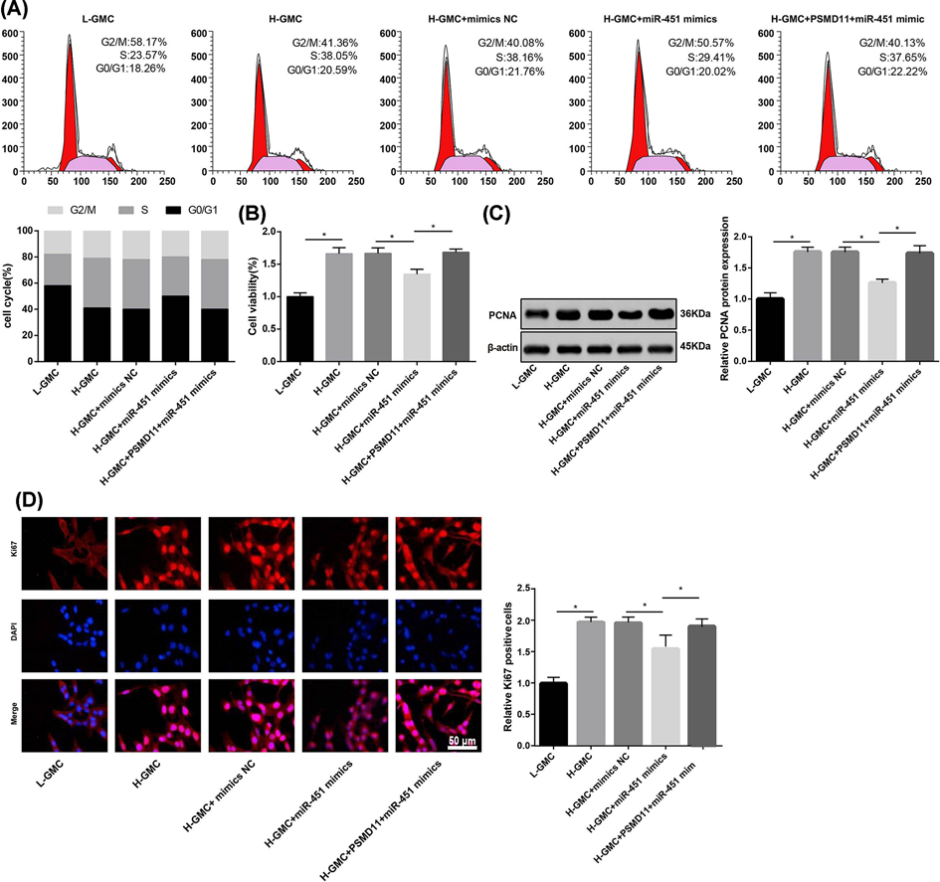
The proliferation of GMCs (**A**) Cell cycle detected by flow cytometry. (**B**) Cell viability detected by MTT assay. (**C**) The expression of PCNA detected by Western blot at the protein level. (**D**) Relative Ki67 positive cells detected by immunofluorescence staining. L-GMCs, GMCs treated with 5.6 mmol/l glucose (normal control); H-GMCs, GMCs treated with 30 mmol/l glucose (high glucose); H-GMCs + mimics NC, H-GMCs transfected with miR-451 mimics negative control; H-GMCs + miR-451 mimics, H-GMCs transfected with miR-451 mimics; H-GMCs + PSMD11 + miR-451 mimics, H-GMCs transfected with pcDNA3.1-PSMD11 and miR-451 mimics. *P<0.05.

The cell cycle and viability of GMCs were detected by flow cytometry and MTT assay, respectively. As shown in Figure 4A, significantly less cells in G_0_/G_1_ phase, and more cells in S phase were observed in H-GMCs (G_0_/G_1_, 20.59%; S, 38.05%) than in L-GMCs (G_0_/G_1_, 18.26%; S, 23.57%) (*P*<0.05). The transfection of miR-451 mimics significantly increased the percentage of cells in G_0_/G_1_ phase (20.02%), and decreased the percentage of cells in S phase (22.22%) (*P*<0.05) (Figure 4A). In addition, the cell viability was significantly higher in H-GMCs (1.663 ± 0.093) than in L-GMCs (1.000 ± 0.060) (*P*<0.05). The transfection of miR-451 mimics significantly decreased the viability of H-GMCs (1.338 ± 0.054) (*P*<0.05) (Figure 4B). Consistent with cell viability, PCNA (a cell proliferation marker) expression, and the number of Ki67 (a cell proliferation marker) positive cells were both significantly increased in H-GMCs (PCNA, 1.757 ± 0.076; Ki67, 1.913 ± 0.055) when compared with L-GMCs (PCNA, 1.000 ± 0.089; Ki67, 1.000 ± 0.103) (*P*<0.05). The up-regulated PCNA and Ki67 in H-GMCs was significantly decreased by the transfection of miR-451 mimics (PCNA, 1.260 ± 0.062; Ki67, 1.403 ± 0.083) (*P*<0.05) (Figure 4C,D). Noteworthily, the effects of miR-451 mimics on the cell cycle (G_0_/G_1_, 22.22%; S, 37.65%), cell viability (1.683 ± 0.056), as well as PCNA and Ki67 expression (PCNA, 1.733 ± 0.123; Ki67, 2.007 ± 0.117) were significantly reversed by the transfection of PSMD11 in miR-451 mimics-transfected H-GMCs (*P*<0.05) (Figure 4A,D).

### Up-regulation of miR-451 inhibited NF-κB activation in H-GMCs

**Figure 5:**
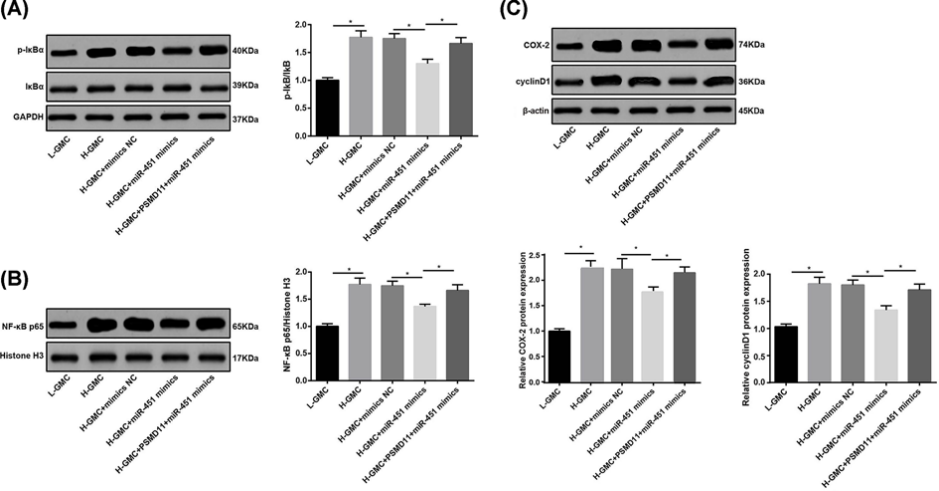
The expression of IκBα, p-IκBα, and NF-κB p65 in GMCs (**A**) The expression of IκBα and p-IκBα detected by Western blot at the protein level. (**B**) The expression of NF-κB p65 detected by Western blot at the protein level. (**C**) The expression of COX-2 and cyclinD1 detected by Western blot at the protein level. L-GMCs, GMCs treated with 5.6 mmol/l glucose (normal control); H-GMCs, GMCs treated with 30 mmol/l glucose (high glucose); H-GMCs + mimics NC, H-GMCs transfected with miR-451 mimics negative control; H-GMCs + miR-451 mimics, H-GMCs transfected with miR-451 mimics; H-GMCs + PSMD11 + miR-451 mimics, H-GMCs transfected with pcDNA3.1-PSMD11 and miR-451 mimics. *P<0.05.

In order to reveal the regulatory mechanisms of miR-451 related to NF-κB signaling, the expression of IκBα, p-IκBα, NF-κB p65, as well as two NF-kB downstream targets COX-2 and cyclinD1 were detected. As shown in Figure 5A,B, the expression of p-IκB/IκB and NF-κB p65 was significantly higher in H-GMCs (p-IκB/IκB, 1.772 ± 0.125; NF-κB p65, 1.764 ± 0.118) than in L-GMCs (p-IκB/IκB, 1.000 ± 0.058; NF-κB p65, 1.000 ± 0.046) (*P*<0.05). The transfection of miR-451 mimics significantly down-regulated p-IκBα and NF-κB p65 in H-GMCs (p-IκB/IκB, 1.315 ± 0.083; NF-κB p65, 1.374 ± 0.048) (*P*<0.05). The down-regulated p-IκBα and NF-κB p65 were recovered by the transfection of PSMD11 in miR-451 mimics-transfected H-GMCs (p-IκB/IκB, 1.662 ± 0.106; NF-κB p65, 1.647 ± 0.114) (*P*<0.05, Figure 5A,B). Consistent results with p-IκB/IκB and NF-κB p65 were also observed on the expression of COX-2 and cyclinD1 by Western blot (Figure 5C).

## Discussion

DN is a serious diabetic complication that may lead to glomerular sclerosis and interstitial fibrosis [19]. Since miRs are closely associated with the regulation of inflammation, miR-targeted therapy exhibits a promising prospect in the treatment of DN [7,20]. MiR-451 is down-regulated in diverse tumors, including non-small-cell lung carcinoma [21], papillary thyroid carcinoma [22], neck squamous cell carcinoma [23], and renal cell carcinoma [24]. Noteworthily, miR-451 is also down-regulated in DN, such as the kidney of DN mice, PBMCs of DN patients, as well as GMCs under high-glucose condition [9,10]. Consistent with previous studies, we found that miR-451 expression was significantly lower in H-GMCs than in L-GMCs. Based on this result, miR-451 was up-regulated in H-GMCs by the transfection of miR-451 mimics, and the specific roles of miR-451 on the inflammation and proliferation of H-GMCs were further analyzed.

DN is a chronic low-grade inflammatory process, which is associated with the activation of pro-inflammatory cytokines [25]. In this study, significantly higher IL-1β, IL-6, and IL-8 levels were observed in H-GMCs than in L-GMCs. These results illustrate that the inflammatory response is enhanced by high-glucose in H-GMCs. Previous studies have proved that pro-inflammatory cytokines are negatively regulated by miR-451 [26,27]. For example, the transfection of miR-451 mimics decreases the levels of TNF-α, IL-1β, and IL-6 in synovial fibroblasts isolated from rheumatoid arthritis patients [26]. Overexpression of miR-451 inhibits microglia-induced release of IL-6, IL-1β, and TNF-α [27]. In this study, the transfection of miR-451 mimics significantly decreased IL-1β, IL-6, and IL-8 levels in H-GMCs. Our findings are just consistent with previous studies, and illustrate that the inflammatory response is relieved by the up-regulation of miR-451 in H-GMCs. In addition, it has been proved that some agents exhibit great potential in the treatment of DN via inhibiting pro-inflammatory cytokines, such as resveratrol [28], ligustrazine [29], notoginsenoside R1 [30], and ellagic acid [15]. We suspect that the up-regulation of miR-451 may protect against DN through relieving inflammatory response.

GMCs, the major constituents of the renal glomerulus are important in mesangial matrix homeostasis, glomerular filtration, and phagocytosis [31]. Excessive proliferation of GMCs is a major contributing factor to DN [32]. In this study, we found that H-GMCs exhibited significantly higher cell viability, as well as PCNA and Ki67 expression than L-GMCs. These phenomena illustrate that the proliferation of GMCs is enhanced by high glucose in H-GMCs. In addition, the transfection of miR-451 mimics significantly decreased the cell viability, and down-regulated PCNA and Ki67 in H-GMCs. These findings are just consistent with a previous study that miR-451 overexpression inhibits the proliferation of GMCs both *in vitro* and *in vivo* [33]. Up-regulation of miR-451 may inhibit the proliferation of H-GMCs by blocking cells in G_0_/G_1_ phase. Besides, previous studies have proved that some therapeutic agents targeting DN are effective in the inhibition of GMCs proliferation, such as corosolic acid [31], betulinic acid [31], and triptolide [32]. We suspect that the up-regulation of miR-451 may ameliorate DN through inhibiting GMCs proliferation.

NF-κB signaling plays an important role in diverse biological processes, such as bipolar spindle assembly [34], vertebrate brain development and function [35], as well as cancer initiation and progression [36]. NF-κB is usually activated in DN, and also closely associated with inflammatory response [13]. In this study, the expression of p-IκBα and NF-κB p65 were significantly higher in H-GMCs than in L-GMCs. It is known that NF-κB p65 is activated by the degradation of IκBα, and then induces inflammatory response by binding to specific promoter of the target inflammatory genes [37,38]. Our findings illustrate that NF-κB is activated by high-glucose in H-GMCs. The activation of NF-κB contributes to the high levels of IL-1β, IL-6, and IL-8 in H-GMCs. In addition, miR-451 has been proved to inhibit pro-inflammatory molecules through negatively regulating NF-κB [9,39]. For example, up-regulation of miR-451 inhibits the NF-κB activity by targeting LMP7, thereby inhibiting the transcription of pro-inflammatory molecules in mesangial cells [9]. Overexpression of miR-451 inhibits the translocation of NF-κB p65 into the nucleus, thereby suppressing palmitate-induced production of proinflammatory cytokines in steatotic cells [39]. In consistent with previous studies, the transfection of miR-451 mimics significantly down-regulated p-IκBα and NF-κB p65 in H-GMCs. Our findings illustrate that the up-regulation of miR-451 may relieve the inflammatory response of H-GMCs via inhibiting the activation of NF-κB. In addition, miR-451 mimics-induced down-regulation of COX-2 and cyclinD1 (two NF-kB downstream targets involved in inflammation) further illustrate that miR-451 up-regulation blocks NF-κB singling in H-GMCs. Previous studies have proved that diverse agents targeting NF-κB singling can ameliorate DN *in vivo*, such as irbesartan [40], timosaponin B-II [41], caffeic acid *para*-nitro phenethyl ester [42], Jiangtang decoction [43], and berberine [44]. For example, irbesartan ameliorates metabolic abnormalities, renal dysfunction, and podocyte injury in type 2 DN mice through suppressing the RANKL-RANK-NF-κB pathway [40]. Timosaponin B-II ameliorates the renal histopathological injury and inflammation in alloxan-induced DN mice [41]. We suspect that miR-451 up-regulation may also contribute to the amelioration of DN *in vivo* through blocking NF-κB signaling. However, the present study is still limited in cellular level, and animal-based studies are needed.

PSMD11 is a 26S proteasome non-ATPase regulatory subunit required for proteasome assembly [17]. A previous study has proved that the knockdown of PSMD13 inhibits the production of proinflammatory mediators in lipopolysaccharide-stimulated BV2 microglia via inhibiting IκBα degradation and NF-κB activation [45]. However, the knowledge on the regulatory roles of PSMD11 on DN is greatly limited. In the present study, PSMD11 was identified as a target of miR-451. In contrast with miR-451, the expression of PSMD11 was significantly higher in H-GMCs than in L-GMCs. The transfection of miR-451 mimics significantly down-regulated PSMD11 in H-GMCs. These results indicate that PSMD11 is negatively regulated by miR-451 in H-GMCs. Since the transfection of PSMD11 could not influence miR-451 expression, PSMD11 may not feedback regulate miR-451 in H-GMCs. In addition, we found that the inhibitory effects of miR-451 mimics on the proliferation, inflammation, and NF-κB activation of H-GMCs were significantly reversed by the transfection of PSMD11. We suspect that PSMD11 may exert similar functions with PSMD13 in H-GMCs. The down-regualtion of PSMD11 may also contribute to the amelioration of DN. However, the roles and regulatory mechanisms of PSMD11 on H-GMCs still need to be studied.

## Conclusion

MiR-451 negatively regulated its target PSMD11. The up-regulation of miR-451 significantly inhibited the inflammation and proliferation of H-GMCs through down-regulating PSMD11 and NF-κB p65. The up-regulation of miR-451 may be a promising therapeutic target for DN.

## Abbreviations

COL1A1: collagen type 1 alpha 1 chain
COX-2: cytochrome c oxidase subunit 2
DLR: dual luciferase reporter
DN: diabetic nephropathy
GAPDH: glyceraldehyde-3-phosphate dehydrogenase
GMC: glomerular mesangial cell
HRP: horseradish peroxidase
IL: interleukin
IκBα: inhibitor of NF-κB α
LMP7: large multifunctional protease 7
miR: microRNA
miR-451: microRNA-451
NF-κB: nuclear factor-κB
OD: optical density
PBMC: peripheral blood mononuclear cell
PBS: phosphate buffer saline
PCNA: proliferating cell nuclear antigen
PSMD11: 26S proteasome non-ATPase regulatory subunit 11
PSMD11-MUT: PSMD11-mutant
PSMD11-WT: PSMD11-wildtype
p-IκBα: phosphorylated IκBα
qRT-PCR: quantitative real-time PCR
RANK: receptor activator of nulear factor κB
RANKL: receptor activator of nulear factor κB ligand

## References

[1] Kolgiri V.and Patil V.W. (2017) Protein carbonyl content: a novel biomarker for aging in HIV/AIDS patients. Braz. J. Infect. Dis. 21, 35–41 10.1016/j.bjid.2016.09.007 27821249 PMC9425472

[2] Han Q., Zhu H., Chen H.and Liu Z. (2017) Non-genetic mechanisms of diabetic nephropathy. Front. Med. 11, 319–332 10.1007/s11684-017-0569-9 28871454

[3] Uwaezuoke S.N. (2017) The role of novel biomarkers in predicting diabetic nephropathy: a review. Int. J. Nephrol. Renovasc. Dis. 10, 221–231 10.2147/IJNRD.S143186 28860837 PMC5566367

[4] Fineberg D., Jandeleit-Dahm K.A.and Cooper M.E. (2013) Diabetic nephropathy: diagnosis and treatment. Nat. Rev. Endocrinol. 9, 713–723 10.1038/nrendo.2013.184 24100266

[5] Rheinberger M., Büttner R.and Böger C.A. (2016) New aspects in prevention and therapy of diabetic nephropathy. Dtsch. Med. Wochenschr. 141, 186 10.1055/s-0041-109591 26841180

[6] Tüfekci K.U., Meuwissen R.L.and Genç S. (2014) The role of microRNAs in biological processes. Methods Mol. Biol. 1107, 15–31 10.1007/978-1-62703-748-8_2 24272429

[7] Wu H., Kong L., Zhou S., Cui W., Xu F., Luo M. et al. (2014) The role of microRNAs in diabetic nephropathy. J. Diabetes Res. 2014, 920134 10.1155/2014/920134 25258717 PMC4165734

[8] Kato M.and Natarajan R. (2015) MicroRNAs in diabetic nephropathy: functions, biomarkers, and therapeutic targets. Ann. N.Y. Acad. Sci. 1353, 72 10.1111/nyas.12758 25877817 PMC4607544

[9] Sun Y., Peng R., Peng H., Liu H., Wen L., Wu T. et al. (2016) miR-451 suppresses the NF-kappaB-mediated proinflammatory molecules expression through inhibiting LMP7 in diabetic nephropathy. Mol. Cell. Endocrinol. 433, 75–86 27264074 10.1016/j.mce.2016.06.004

[10] Jiang W.H., Sun Y., Peng R., Peng H.M.and Zhang Z. (2018) miR-451 inhibits inflammatory responses in glomerular mesangial cells by targeting Psmb8 in diabetic nephropathy mice. Chin. J. Pathophysiol. 34, 494–499

[11] Mohan A., Singh R.S., Kumari M., Garg D., Upadhyay A., Ecelbarger C.M. et al. (2016) Urinary exosomal microRNA-451-5p is a potential early biomarker of diabetic nephropathy in rats. PLoS ONE 11, e0154055– 10.1371/journal.pone.0154055 27101382 PMC4839711

[12] Huang W., Ling X.U., Zhou X.Q., Yong X.U.and Endocrinology D.O. (2014) Effects of SUMOylation on IKKγ/NF-κB signaling in cultured rat glomerular mesangial cells treated with high glucose. Chin. J. Pathophysiol. 30, 538–542

[13] Mezzano S., Aros C., Droguett A., Eugenia Burgos M., Ardiles L., Flores C. et al. (2004) NF-kappaB activation and overexpression of regulated genes in human diabetic nephropathy. Nephrol. Dial. Transplant. 19, 2505–2512 10.1093/ndt/gfh207 15280531

[14] Das K.and Ghosh M. (2017) Structured DAG oil ameliorates renal injury in streptozotocin-induced diabetic rats through inhibition of NF-κB and activation of Nrf2 pathway. Food Chem. Toxicol. 100, 225 10.1016/j.fct.2016.12.033 28025123

[15] Ahad A., Ganai A.A., Mujeeb M.and Siddiqui W.A. (2014) Ellagic acid, an NF-κB inhibitor, ameliorates renal function in experimental diabetic nephropathy. Chem. Biol. Interact. 219, 64–75 10.1016/j.cbi.2014.05.011 24877639

[16] Soetikno V., Sari F.R., Veeraveedu P.T., Thandavarayan R.A., Harima M., Sukumaran V. et al. (2011) Curcumin ameliorates macrophage infiltration by inhibiting NF-κB activation and proinflammatory cytokines in streptozotocin induced-diabetic nephropathy. Nutr. Metab. 8, 35–35 10.1186/1743-7075-8-35 PMC3123175 21663638

[17] Vilchez D., Boyer L., Morantte I., Lutz M., Merkwirth C., Joyce D. et al. (2012) Increased proteasome activity in human embryonic stem cells is regulated by PSMD11. Nature 489, 304–308 10.1038/nature11468 22972301 PMC5215918

[18] Livak K.J.and Schmittgen T.D. (2001) Analysis of relative gene expression data using real-time quantitative PCR and the 2(-Delta Delta C(T))method. Methods 25, 402–408 10.1006/meth.2001.1262 11846609

[19] Tang S., Gao C., Long Y., Huang W., Chen J., Fan F. et al. (2017) Maresin 1 mitigates high glucose-induced mouse glomerular mesangial cell injury by inhibiting inflammation and fibrosis. Mediators Inflamm. 2017, 1–11 10.1155/2017/2438247 PMC5274668 28182085

[20] Chen L.C., Conos S.A., Unal B.and Tergaonkar V. (2018) Noncoding RNAs: master regulators of inflammatory signaling. Trends Mol. Med. 24, 66–84 10.1016/j.molmed.2017.11.003 29246760

[21] Wang X.C., Tian L.L., Jiang X.Y., Wang Y.Y., Li D.G., She Y. et al. (2011) The expression and function of miRNA-451 in non-small cell lung cancer. Cancer Lett. 311, 203–209 10.1016/j.canlet.2011.07.026 21875770

[22] Zhang M., Wu W., Gao M.and Fei Z. (2017) MicroRNA-451 as a prognostic marker for diagnosis and lymph node metastasis of papillary thyroid carcinoma. Cancer Biomarkers 19, 437–445 10.3233/CBM-170059 28582849 PMC13020739

[23] Wang H., Zhang G., Wu Z., Lu B., Yuan D., Li X. et al. (2015) MicoRNA-451 is a novel tumor suppressor via targeting c-myc in head and neck squamous cell carcinomas. J. Cancer Res. Ther. c216–c221 10.3892/mmr.2014.2149 26506880

[24] Tang Y., Wan W., Wang L., Ji S.and Zhang J. (2015) microRNA-451 inhibited cell proliferation, migration and invasion through regulation of MIF in renal cell carcinoma. Int. J. Clin. Exp. Pathol. 8, 15611–15621 26884830 PMC4730043

[25] Jun W.and Hirofumi M. (2013) Inflammation and the pathogenesis of diabetic nephropathy. Clin. Sci. 124, 139–152 10.1042/CS20120198 23075333

[26] Wang Z.C., Lu H., Zhou Q., Yu S.M., Mao Y.L., Zhang H.J. et al. (2015) MiR-451 inhibits synovial fibroblasts proliferation and inflammatory cytokines secretion in rheumatoid arthritis through mediating p38MAPK signaling pathway. Int. J. Clin. Exp. Pathol. 8, 14562–14567 26823778 PMC4713564

[27] Sun X.and Zhang H. (2018) miR-451 elevation relieves inflammatory pain by suppressing microglial activation-evoked inflammatory response via targeting TLR4. Cell. Tissue Res. 10.1007/s00441-018-2898-7 30069596

[28] Chih-Chun C., Chieh-Yu C., Yang-Tzu W., Jiung-Pang H., Tzung-Hai Y.and Li-Man H. (2011) Resveratrol retards progression of diabetic nephropathy through modulations of oxidative stress, proinflammatory cytokines, and AMP-activated protein kinase. J. Biomed. Sci. 18, 47–47 10.1186/1423-0127-18-47 21699681 PMC3150248

[29] Yang H.and Wu S. (2018) Ligustrazine attenuates renal damage by inhibiting endoplasmic reticulum stress in diabetic nephropathy by inactivating MAPK pathways. Rsc. Adv. 8, 21816–21822 10.1039/C8RA01674G PMC9080983 35541710

[30] Huang G., Lv J., Li T., Huai G., Li X., Xiang S. et al. (2016) Notoginsenoside R1 ameliorates podocyte injury in rats with diabetic nephropathy by activating the PI3K/Akt signaling pathway. Int. J. Mol. Med. 38, 1179–1189 10.3892/ijmm.2016.2713 27571993 PMC5029967

[31] Wawrzyniak P., Wawrzyniak M., Wanke K., Sokolowska M., Bendelja K., Rückert B. et al. (2016) Regulation of bronchial epithelial barrier integrity by type 2 cytokines and histone deacetylases in asthma. J. Allergy Clin. Immunol. 139, 93 10.1016/j.jaci.2016.03.050 27312821

[32] Fei H., Mei X., Chang Y., Li X., Yang Y., Bei S. et al. (2017) Triptolide suppresses glomerular mesangial cell proliferation in diabetic nephropathy is associated with inhibition of PDK1/Akt/mTOR pathway. Int. J. Biol. Sci. 13, 1266–1275 10.7150/ijbs.20485 29104493 PMC5666525

[33] Zheng Z., Xiaomei L., Songtao D., Junxia C., Tao C., Xin C. et al. (2012) MicroRNA-451 regulates p38 MAPK signaling by targeting of Ywhaz and suppresses the mesangial hypertrophy in early diabetic nephropathy. FEBS Lett. 586, 20–26 10.1016/j.febslet.2011.07.042 21827757

[34] Irelan J.T., Murphy T.J., DeJesus P.D., Teo H., Xu D., Gomez-Ferreria M.A. et al. (2007) A role for IkB kinase 2 in bipolar spindle assembly. Proc. Natl. Acad. Sci. U.S.A. 104, 16940–16945 10.1073/pnas.0706493104 17939994 PMC2040438

[35] Ang H.L.and Tergaonkar V. (2007) Notch and NFκB signaling pathways: do they collaborate in normal vertebrate brain development and function. Bioessays 29, 1039–1047 10.1002/bies.20647 17876798

[36] Yu P., Muthu S., Lu F., Frank A., Gautam S.and Vinay T. (2018) Evidence for the involvement of the master transcription factor NF-κB in cancer initiation and progression. Biomedicine 6, 82– 10.3390/biomedicines6030082 PMC6163404 30060453

[37] Frank C., Smith E.L.and Carmody R.J. (2016) The regulation of NF-κB subunits by phosphorylation. Cells 5, 12 10.3390/cells5010012 PMC4810097 26999213

[38] Correa R.G., Matsui T., Tergaonkar V., Rodriguezesteban C., Izpisuabelmonte J.C.and Verma I.M. (2005) Zebrafish IkappaB kinase 1 negatively regulates NF-kappaB activity. Curr.t Biol. 15, 1291 10.1016/j.cub.2005.06.023 16051172

[39] Hur W., Lee J.H., Kim S.W., Kim J.H., Si H.B., Kim M. et al. (2015) Downregulation of microRNA-451 in non-alcoholic steatohepatitis inhibits fatty acid-induced proinflammatory cytokine production through the AMPK/AKT pathway. Int. J. Biochem. Cell Biol. 64, 265–276 10.1016/j.biocel.2015.04.016 25957914

[40] Xiao-Wen C., Xiao-Yan D., Yu-Xian W., Jian-Cheng W., Wen-Ting L., Wen-Jing C. et al. (2016) Irbesartan ameliorates diabetic nephropathy by suppressing the RANKL-RANK-NF-κB pathway in type 2 diabetic db/db mice. Mediators Inflamm. 2016, 1405924 26880862 10.1155/2016/1405924 PMC4736580

[41] Fei L., Yong-Liang Y., Chang-Run G., Ling-Ling C., Shi-Xia R., Chun-Feng Z. et al. (2015) Timosaponin B-II ameliorates diabetic nephropathy via TXNI P, mTOR, and NF-&kappa;B signaling pathways in alloxan-induced mice. Drug Des. Dev. Ther. 9, 6247–6258 10.2147/DDDT.S96435 PMC4669930 26664046

[42] Wang X., Li D., Lu F., Xiao Q.and Li Z. (2017) CAPE-pNO2 ameliorated diabetic nephropathy through regulating the Akt/NF-κB/ iNOS pathway in STZ-induced diabetic mice. Oncotarget 8, 114506–114525 10.18632/oncotarget.23016 29383098 PMC5777710

[43] Hong J.N., Li W.W., Wang L.L., Guo H., Jiang Y.and Gao Y.-J. (2017) Jiangtang decoction ameliorate diabetic nephropathy through the regulation of PI3K/Akt-mediated NF-κB pathways in KK-Ay mice. Chin. Med. 12, 13 10.1186/s13020-017-0134-0 28529539 PMC5437490

[44] Zhu L., J H., R Y., L X.and W P. (2018) Berberine ameliorates diabetic nephropathy by inhibiting TLR4/NF-κB pathway. Biol. Res. 51, 9 10.1186/s40659-018-0157-8 29604956 PMC5878418

[45] Wei B., Lihong Z., Zhifen Z., Xiuna J., Yanran L., Li G. et al. (2014) Investigations into the role of 26S proteasome non-ATPase regulatory subunit 13 in neuroinflammation. NeuroImmunoModulation 21, 331–337 10.1159/000357811 24642793

